# A genome-wide association study reveals that epistasis underlies the pathogenicity of *Pectobacterium*

**DOI:** 10.1128/spectrum.01764-23

**Published:** 2023-09-15

**Authors:** Changlong Chen, Shu Che, Zhou Dong, Jiayi Sui, Yu Tian, Yanyan Su, Meng Zhang, Wangwang Sun, Jiaqin Fan, Jianbo Xie, Hua Xie

**Affiliations:** 1 Institute of Biotechnology, Beijing Academy of Agriculture and Forestry Sciences, Beijing, China; 2 Department of Plant Pathology, Nanjing Agricultural University, Nanjing, China; 3 EVision Technology (Beijing) Co. Ltd, Beijing, China; 4 State Key Laboratory of Tree Genetics and Breeding, College of Biological Sciences and Technology, Beijing Forestry University, Beijing, China; University of Minnesota Twin Cities, St. Paul, Minnesota, USA

**Keywords:** *Pectobacterium *spp., genome-wide association studies (GWAS), pathogenicity, epistasis, artificial intelligence

## Abstract

*Pectobacterium* spp. are important bacterial pathogens that cause soft rot symptoms in various crops. However, their mechanism of pathogenicity requires clarity to help control their infections. Here, genome-wide association studies (GWAS) were conducted by integrating genomic data and measurements of two phenotypes (virulence and cellulase activity) for 120 various *Pectobacterium* strains in order to identify the genetic basis of their pathogenicity. An artificial intelligence-based software program was developed to automatically measure lesion areas on Chinese cabbage, thereby facilitating accurate and rapid data collection for virulence phenotypes for use in GWAS analysis. The analysis discovered 428 and 158 loci significantly associated with *Pectobacterium* virulence (lesion area) and cellulase activity, respectively. In addition, 1,229 and 586 epistasis loci pairs were identified for the virulence and cellulase activity phenotypes, respectively. Among them, the AraC transcriptional regulator exerted epistasis effects with another three nutrient transport-related genes in pairs contributing to the virulence phenotype, and their epistatic effects were experimentally confirmed for one pair with knockout mutants of each single gene and double gene. This study consequently provides valuable insights into the genetic mechanism underlying *Pectobacterium* spp. pathogenicity.

**Importance:**

Plant diseases and pests are responsible for the loss of up to 40% of food crops, and annual economic losses caused by plant diseases reach more than $220 billion. Fighting against plant diseases requires an understanding of the pathogenic mechanisms of pathogens. This study adopted an advanced approach using population genomics integrated with virulence-related phenotype data to investigate the genetic basis of *Pectobacterium* spp., which causes serious crop losses worldwide. An automated software program based on artificial intelligence was developed to measure the virulence phenotype (lesion area), which greatly facilitated this research. The analysis predicted key genomic loci that were highly associated with virulence phenotypes, exhibited epistasis effects, and were further confirmed as critical for virulence with mutant gene deletion experiments. The present study provides new insights into the genetic determinants associated with *Pectobacterium* pathogenicity and provides a valuable new software resource that can be adapted to improve plant infection measurements.

## INTRODUCTION

*Pectobacterium* spp. are responsible for plant soft rot diseases worldwide. They significantly impact global food security due to their ability to infect diverse plant hosts, including economically important vegetables, fruits, and ornamental plants, in the field and also during postharvest transit, storage, and marketing (1 – 3). It is well known that *Pectobacterium* spp. produce plant cell wall-degrading enzymes (PCWDEs) like cellulase that dissolve plant tissues and cause a wet and often foul-smelling rot of plant organs (4, 5). Once initiated, infection symptoms rapidly develop. Indeed, inoculated tubers or storage roots rot within 2 to 3 days, and infected plants die within a few hours of initial wilting symptoms under favorable conditions (5).

The most distinctive feature of soft rot pathogenesis is the simultaneous production of abundant PCWDEs like cellulases, pectinases, hemicellulases, and proteinases that degrade plant tissues to enable successful infection (6, 7). Comparative genomic analyses have shown that PCWDEs are conserved among *Pectobacterium* species (8, 9). Consequently, factors other than PCWDEs may be responsible for differences in disease severity between different *Pectobacterium* species and strains (6). Genomic analyses have indicated that some pathogenic factors, including flagellin, siderophores, polysaccharides, protein secretion systems, and regulatory factors, vary among *Pectobacterium* species (8). However, their influences on virulence variation and whether other factors may contribute to virulence remain unresolved. Meanwhile, gene-by-gene interactions (or epistasis) underlying virulence are largely unknown for *Pectobacterium*.

Genome-wide association studies (GWAS) are a powerful approach to identifying the genetic basis of phenotypic traits by leveraging variation that exists within natural populations and were first applied to investigate human diseases (10 – 13). The method has become a popular approach to evaluating the genetic mechanisms underlying human diseases as well as the complex traits of animals and plants. The approach has also been recently applied to understand bacterial genetic characteristics. For example, the factors responsible for host (cattle and chickens) adaptations of *Campylobacter* were identified using GWAS in 2013 (14). Several investigations of GWAS have subsequently evaluated genome variation associated with important bacterial phenotypes like host adaptability, drug resistance, and virulence (15 – 20). However, the use of GWAS to investigate the virulence of plant bacterial pathogens has only rarely been reported.

Phenotypic accuracy is a critical consideration for the analysis of GWAS. Phenotyping errors introduced by the combination of inconsistent methods used to diagnose disease and the application of imprecise measurement methods can reduce the discovery potential of GWAS (21). In plant pathogen research, lesion size resulting from plant pathogens is generally an important index of pathogen virulence. Lesion size is typically obtained by measuring the maximum lengths and widths to obtain an approximate area based on formulas specific to lesion shapes (22). Nevertheless, this method suffers from large errors and is time-consuming. A more accurate method for determining lesion size is the grid method (23), although this technique is also time- and labor-consuming. In addition, increased numbers of measurements can lead to less accuracy due to operator fatigue. The continuous development of computer graphics and digital technology in recent years has led to increased use of image analysis systems to measure leaf lesion areas (24 – 28). Commonly used software programs for analyzing plant diseases primarily include Assess (American Society of Plant Pathology, USA), ImageJ, and Adobe Photoshop (Adobe, USA), among others. However, they are also time-consuming and difficult to use. In recent years, artificial intelligence has become increasingly developed, and a deep learning approach to analyzing the phenomics of plant diseases is also highly effective for evaluating plant disease severity (29). However, such techniques have been minimally implemented in traditional plant disease studies due to the relative inaccessibility of the software programs. Consequently, a simple and convenient method to analyze lesion areas is needed to advance plant disease studies.

Here, the genetic basis of variation in the virulence of *Pectobacterium* spp. was evaluated with GWAS by investigating the genomic sequences of 120 *Pectobacterium* isolates and their respective virulence-related phenotypes. The underlying epistasis for virulence was predicted and experimentally confirmed. A user-friendly and computationally efficient software program “Lesionsurvey” was also developed to measure lesion areas in <10 s. This study consequently provides new insights into *Pectobacterium* pathogenicity via cutting-edge research approaches in genomics and artificial intelligence.

## RESULTS

### Genotyping and phenotyping of 120 *Pectobacterium* strains

**FIG 1:**
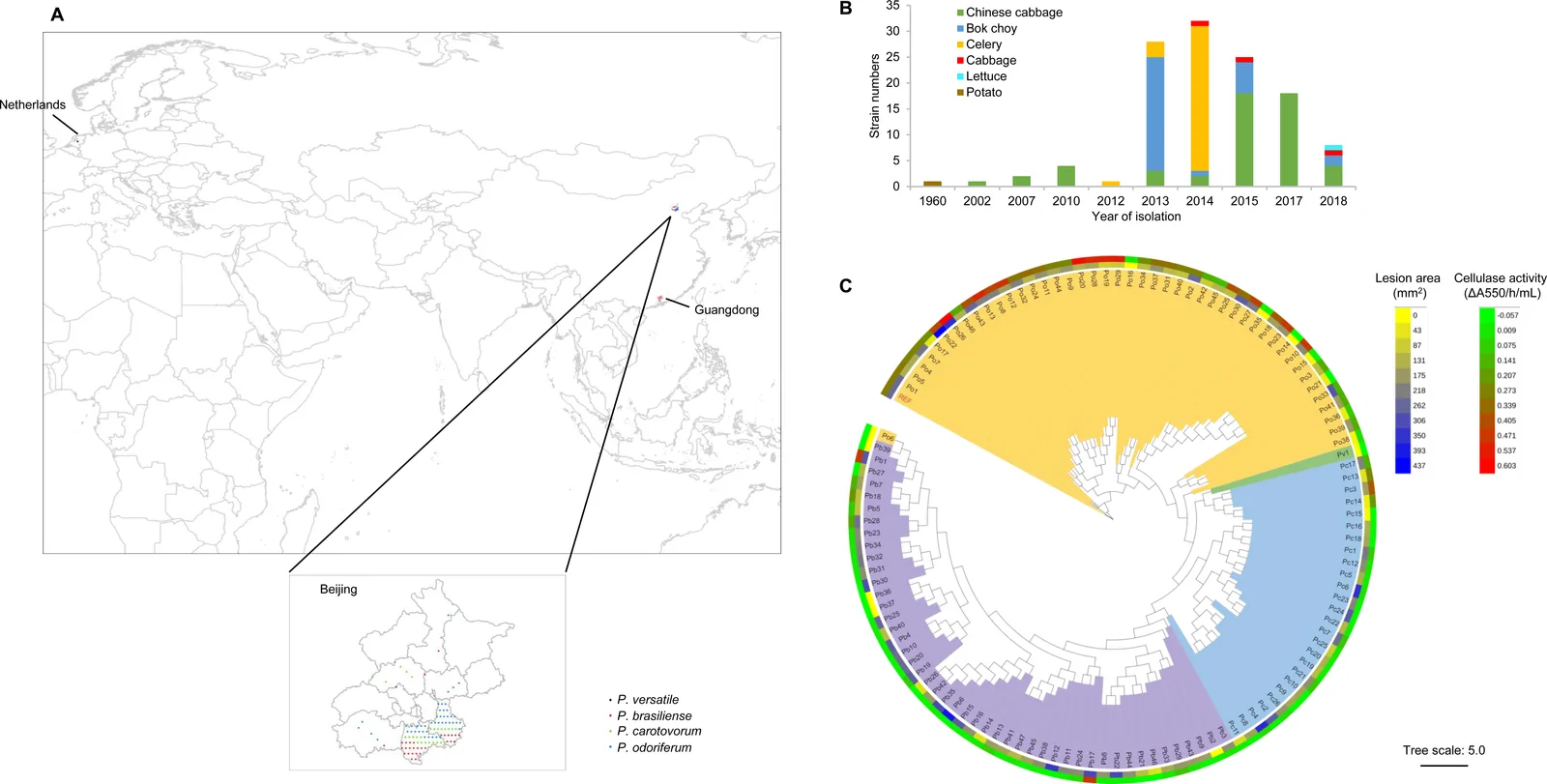
Sampling distribution and genetic structures of 120 *Pectobacterium* strains. (**A**) Geographic distribution of the 120 *Pectobacterium* strains. Each strain isolation point is indicated by a circle. Beijing’s dense collections are magnified. Circles with different colors indicate different species of *Pectobacterium*, as indicated by the legend. (**B**) Distribution of temporal sampling of the 120 *Pectobacterium* strains. The distribution of host origins for the strains is also shown. (**C**) Clustering of strains based on whole-genome SNPs, using Po1 (Po BCS7) as the reference strain (REF). Strains of *P. carotovorum*, *P. brasiliense*, *P. odoriferum*, and *P. versatile* are colored blue, purple, yellow, and green, respectively. The two external circles are gradient plots showing phenotype data for lesion areas (from yellow to blue) and cellulase activity (from green to red) for each strain.

To identify the genetic determinants of *Pectobacterium* that contribute to virulence (i.e., lesion production) and their associated cellulase activity, GWAS were conducted using the whole-genome sequences of 120 *Pectobacterium* strains collected from different districts of Beijing, Guangdong, and the Netherlands (Fig. 1A; Table S1). Strains were isolated from six districts of Beijing, including Tongzhou, Daxing, Changping, Shunyi, Huairou, and Fangshan. The strains were collected between 1960 and 2018, with most collected from 2013 to 2018. A total of 28, 32, 25, 18, and 8 strains were isolated in 2013, 2014, 2015, 2017, and 2018, respectively. The 120 *Pectobacterium* strains were isolated from 6 host plants, including Chinese cabbage (52 strains), bok choy (31 strains), celery (32 strains), cabbage (3 strains), lettuce (1 strain), and potato (1 strain) (Fig. 1B). Except for the single strain associated with *P. versatile*, the other 119 strains comprised 3 *Pectobacterium* species, including *P. carotovorum* (26 strains), *P. brasiliense* (47 strains), and *P. odoriferum* (46 strains). Mapping against the reference genome of Po BCS7 (CP009678.1) and subsequent single nucleotide polymorphism (SNP) identification revealed a total of 230,289 SNPs and 4,506 indels. A phylogenetic tree constructed using the SNP profiles revealed that the strains belonged to three separate species of *Pectobacterium* (Fig. 1C).

**FIG 2:**
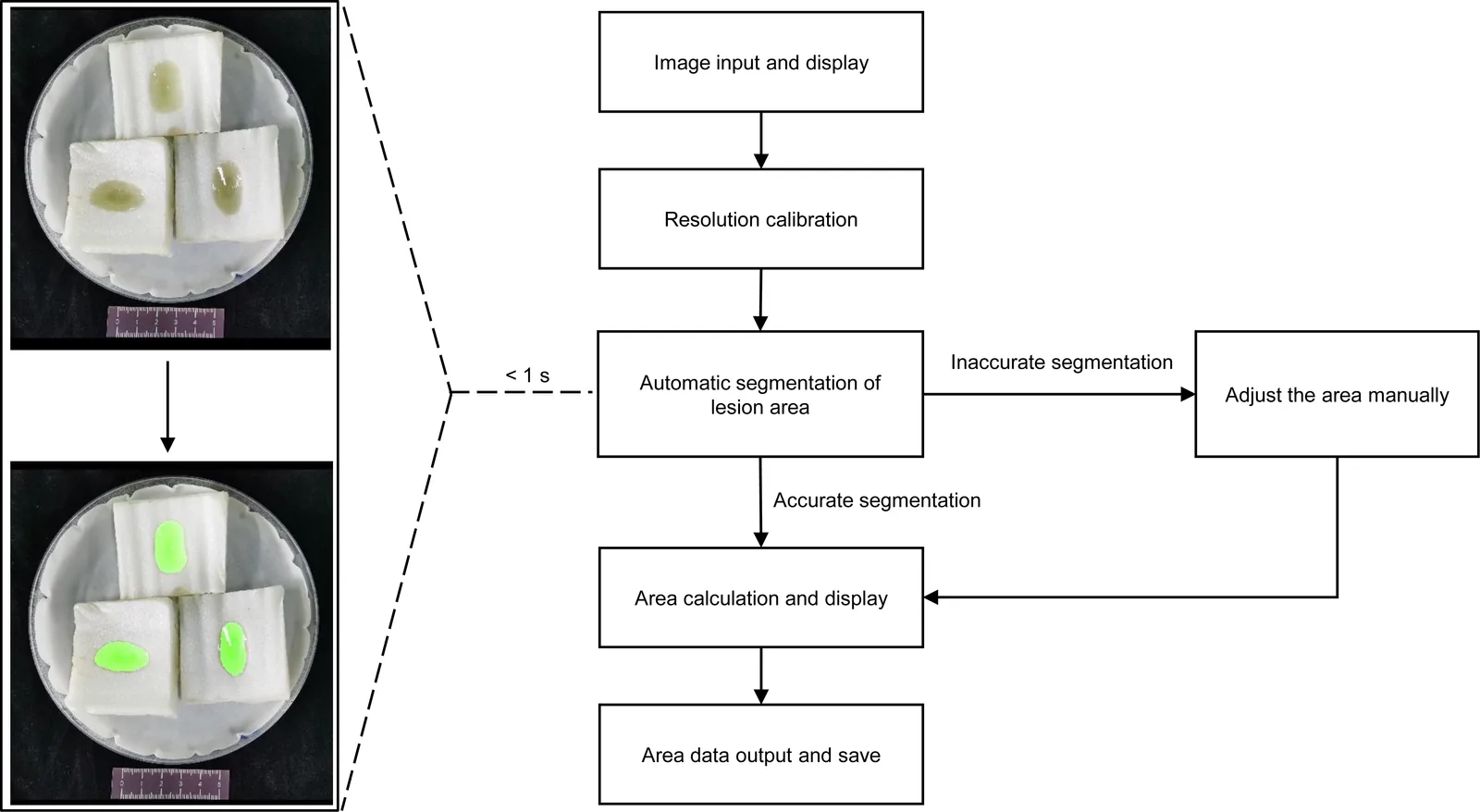
Workflow for automated measurement of lesion areas in Chinese cabbage caused by soft rot disease using the Lesionsurvey software program developed in this study. The whole process can be performed in <10 s.

Cellulases secreted by *Pectobacterium* play important roles in pathogenicity (6). Consequently, virulence and cellulase activity were evaluated using the 120 *Pectobacterium* strains (Table S1). Virulence was identified by lesion areas caused by the strains on Chinese cabbage *in vitro*. The software program developed in this study was employed to rapidly, accurately, and automatically collect lesion area data for numerous *Pectobacterium* strains. Indeed, accurate soft rot area measurements for each experimental image were obtained in <10 s (Fig. 2). In the core module of this program, automatic segmentation of the lesion area takes <1 s. Cellulase activity was also assessed using an enzymatic assay based on absorbance at 550 nm using the culture supernatant from each culture and with cellulase as the substrate.

### Weak correlation between cellulase activity and lesion area produced by *Pectobacterium*

**FIG 3:**
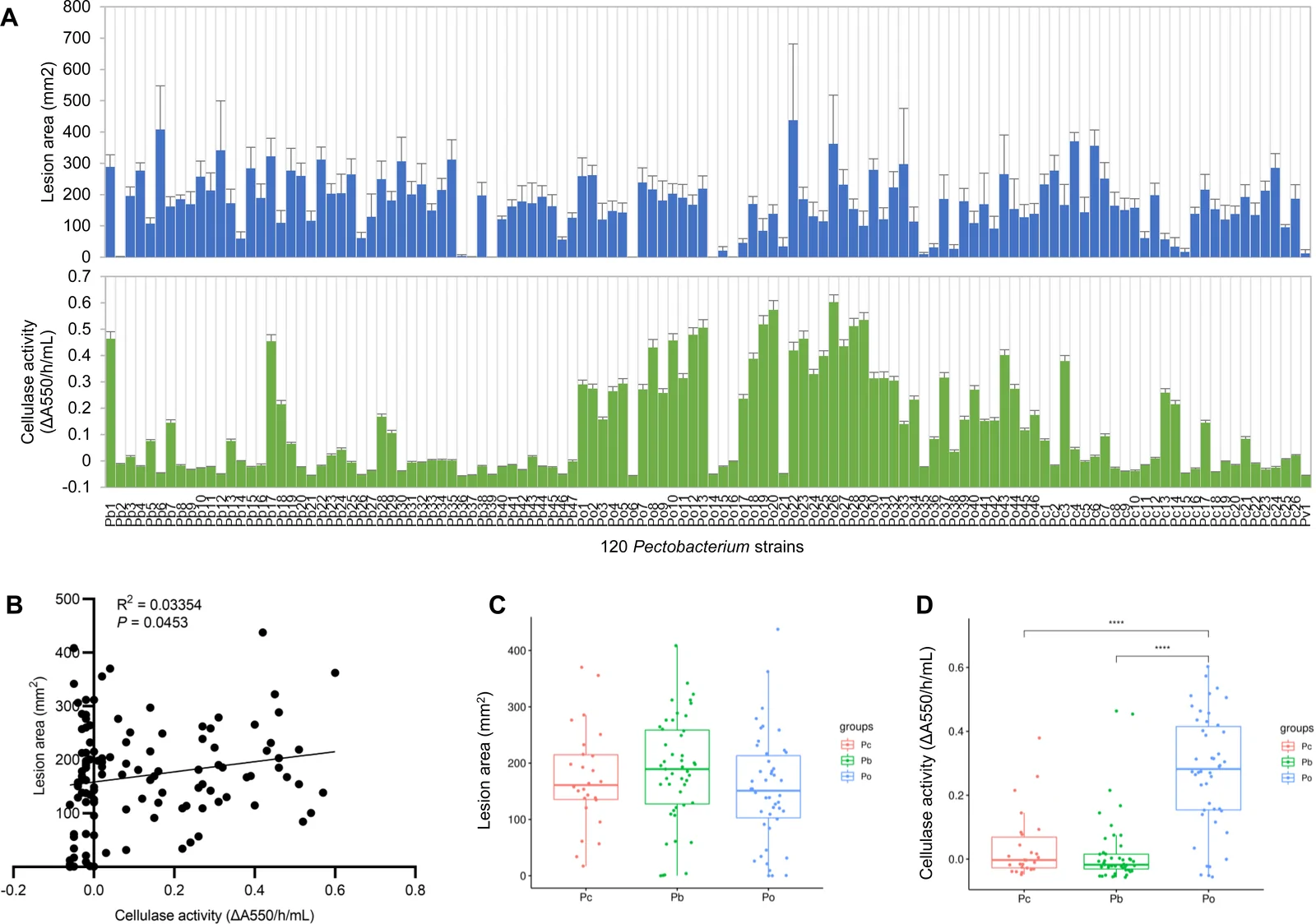
Phenotype data for *Pectobacterium* strains and populations of this study. (**A**) Lesion area (*n* = 6–9) and cellulase activity (*n* = 3) data for the 120 *Pectobacterium* strains of the study. Each column shows the mean ± SE for the strain. (**B**) Correlations between phenotypes of lesion area and cellulase activity. (**C and D**) Comparison of lesion area (**C**) and cellulase activity (**D**) among the three *Pectobacterium* population groups: *P. carotovorum* (*n* = 26), *P. brasiliense* (*n* = 47), and *P. odoriferum* (*n* = 46). ****: *P* < 0.0001 (Student’s *t*-test).

To demonstrate if cellulase activity was associated with *Pectobacterium* virulence, the two phenotypes were considered together (Fig. 3A). Correlation analysis was subsequently conducted for lesion area and cellulase activity, revealing a weak but statistically significant correlation (*R* ^2^ = 0.03354, *P* = 0.0453) (Fig. 3B). Comparison of the two phenotypes among the three *Pectobacterium* species (*P. carotovorum*, *P. brasiliense*, and *P. odoriferum*) revealed a lack of significant difference in lesion area, while *P. odoriferum* exhibited significantly higher cellulase activity (*P <* 0.0001) than *P. carotovorum* and *P. brasiliense* (Fig. 3C and D). These differences were also reflected in the phylogenetic analysis of the *Pectobacterium* strains, with no obvious correlation between evolutionary relationship and lesion area phenotype, while higher cellulase activity was unique to the *P. odoriferum* strains (Fig. 1C).

### Whole-genome screening to identify loci associated with phenotypes

**FIG 4:**
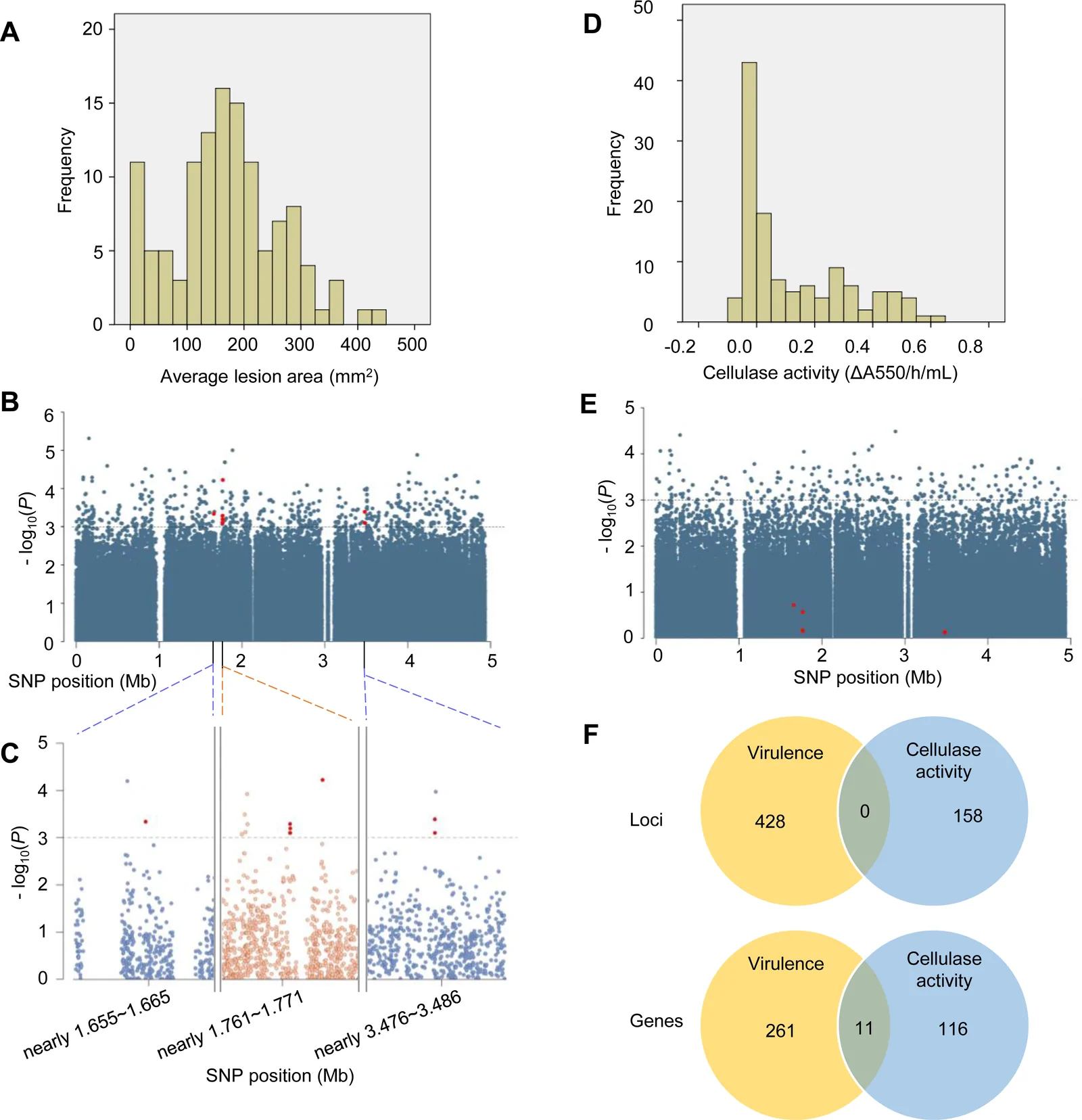
Genome-wide association analysis of virulence (lesion area) and cellulase activity of *Pectobacterium* strains. (**A**) Distribution of average lesion areas across 120 *Pectobacterium* strains. (**B**) Manhattan plot of virulence (lesion area) across the entire population. (**C**) Local Manhattan plots of 5 kb up- and downstream of the epistatic loci 1659628, 1765877, and 3481230, respectively. (**D**) Distribution of cellulase activity for the 120 *Pectobacterium* strains. (**E**) Manhattan plot of cellulase activity across the entire population. (**F**) Venn diagram showing shared and unique significantly associated loci and corresponding genes identified by GWAS for the virulence (lesion area) and cellulase activity phenotypes. The horizontal dashed lines in B, C, and E show the statistical significance threshold (−log_10_(*P*) = 3). Red dots in B, C, and E indicate the seven epistatic loci of virulence that were focused upon: 1659628, 1765877, 1765891, 1765892, 1768194, 3481230, and 3481234.

**FIG 5:**
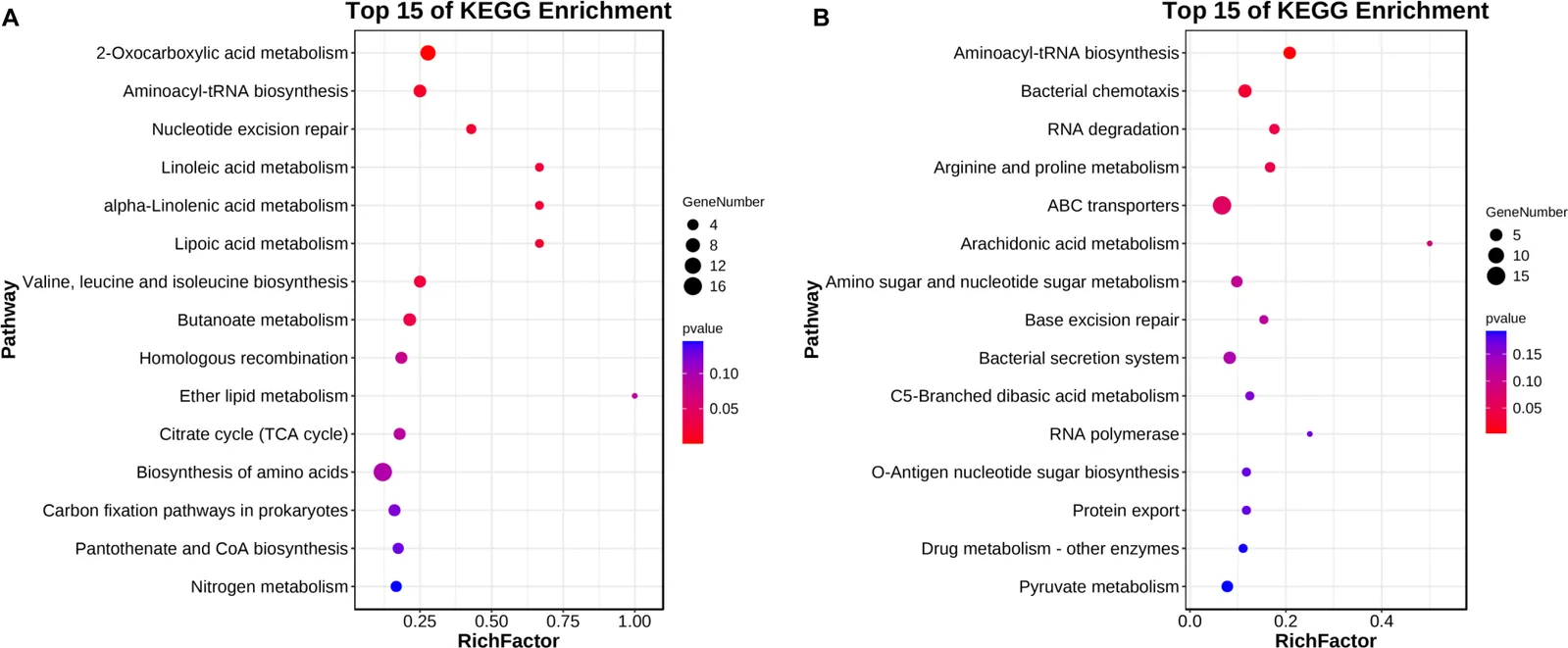
The top 15 pathways enriched by KEGG analysis of genes identified by GWAS associated with lesion area (**A**) and cellulase activity (**B**).

**FIG 6:**
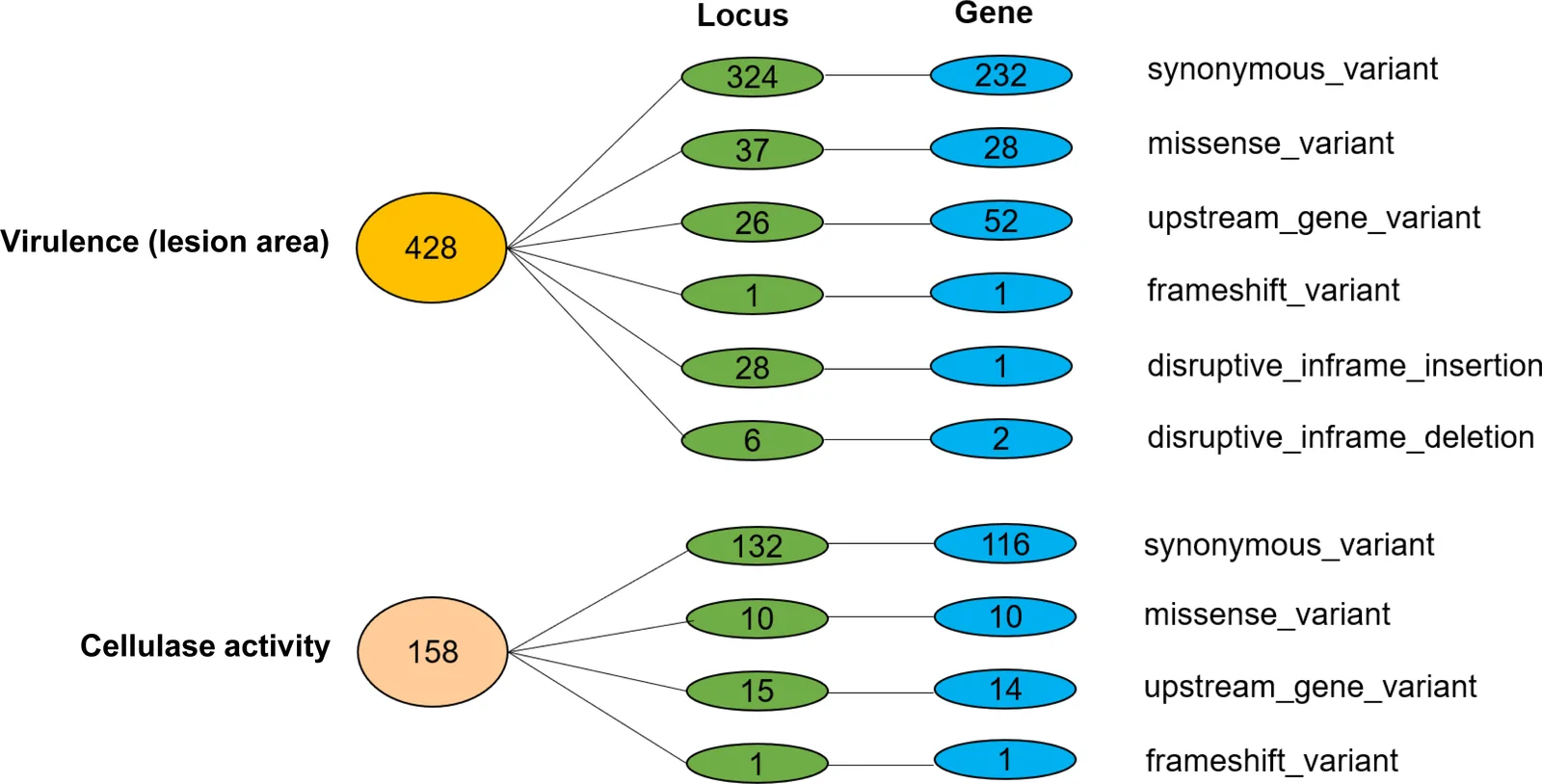
Numbers of different variation types of significant loci identified by GWAS for the virulence (lesion area) and cellulase activity phenotypes.

The lesion area phenotype data exhibited a normal distribution, while the cellulase activity data did not (Fig. 4A and D). Consequently, the former could be directly used for subsequent analysis of GWAS after eliminating individual outliers, while the latter was subjected to COX-BOX conversion before GWAS analysis. GWAS analysis was conducted using data for the two phenotypes and a total of 234,795 SNP markers for *Pectobacterium*. A total of 428 and 158 statistically significant (*P* < 0.001) loci were identified that were correlated with virulence (lesion area) and cellulase activity, respectively (Fig. 4B and E; Tables S2 and S3). After annotation, a total of 272 and 127 genes were identified that were associated with lesion area (virulence) and cellulase activity, respectively (Tables S2 and S3). Kyoto Encyclopedia of Genes and Genomes (KEGG) enrichment analysis indicated that the pathways particularly associated with lesion area included 2-oxocarboxylic acid metabolism, aminoacyl-tRNA biosynthesis, nucleotide excision repair, linoleic acid metabolism, and alpha-linolenic acid metabolism (Fig. 5A). In addition, pathways enriched among genes associated with cellulase activity included aminoacyl-tRNA biosynthesis, bacterial chemotaxis, RNA degradation, arginine and proline metabolism, and ATP-binding cassette (ABC) transporters (Fig. 5B). Among the top 15 pathways enriched by KEGG analysis, the aminoacyl-tRNA biosynthesis pathway is associated with both lesion area and cellulase activity (Fig. 5). Loci were not shared by these two phenotypes, while 11 genes were correlated to both phenotypes, including the genes BCS7_RS05405 (pectate lyase), BCS7_RS15795 (peptidase M4 family protein), BCS7_RS19775 (multispecies: elongation factor Tu), BCS7_RS00020 [DNA topoisomerase (ATP-hydrolyzing) subunit B], and BCS7_RS00700 (DMT family transporter) (Tables S2 and S3). Overall, the genetic analyses revealed a weak correlation between the phenotypes of lesion area (virulence) and cellulase activity. The types of mutations in the identified locus from the GWAS analysis were evaluated for each phenotype (Fig. 6). Synonymous variants comprised most of the mutations in these loci, followed by upstream gene variants and missense variants.

### Epistasis loci are associated with phenotypes

Epistasis, or the interaction between genes, greatly contributes to the control of complex inheritance (30). Putative epistasis loci were consequently predicted for each of the two phenotypes. A total of 1,229 pairs of epistasis loci were identified in total for the virulence phenotype, in addition to 586 for cellulase activity (Tables S4 and S5). Missense variants were identified from the putative epistasis loci, revealing 17 and 2 pairs for virulence and cellulase activity, respectively (Tables S6 and S7). Among the predicted epistasis loci, six loci pairs were of particular interest, which corresponded to three pairs of genes: BCS7_RS07420 (AraC family transcriptional regulator) and BCS7_RS07925 (TonB-dependent receptor); BCS7_RS07420 (AraC family transcriptional regulator) and BCS7_RS07930 (iron-siderophore ABC transporter substrate-binding protein); and BCS7_RS07420 (AraC family transcriptional regulator) and BCS7_RS15950 (multispecies: iron ABC transporter permease) (Table S6; Table 1). The results showed that the AraC family transcriptional regulator exerted epistasis effects with TonB-dependent receptors and ion ABC transporter-related proteins.

**TABLE 1:** Selected epistatic loci identified for the virulence phenotype

No.	Position pair	Gene pair	Gene locus and annotation
1	1659628_1765877	BCS7_RS07420_BCS7_RS07925	BCS7_RS07420: AraC family transcriptional regulator; BCS7_RS07925: TonB-dependent receptor
2	1659628_1765891	BCS7_RS07420_BCS7_RS07925	
3	1659628_1765892	BCS7_RS07420_BCS7_RS07925	
4	1659628_1768194	BCS7_RS07420_BCS7_RS07930	BCS7_RS07420: AraC family transcriptional regulator; BCS7_RS07930: ABC transporter substrate-binding protein
5	1659628_3481230	BCS7_RS07420_BCS7_RS15950	BCS7_RS07420: AraC family transcriptional regulator; BCS7_RS15950: multispecies: iron ABC transporter permease
6	1659628_3481234	BCS7_RS07420_BCS7_RS15950	

### Phenotypes and validation of epistasis loci

**FIG 7:**
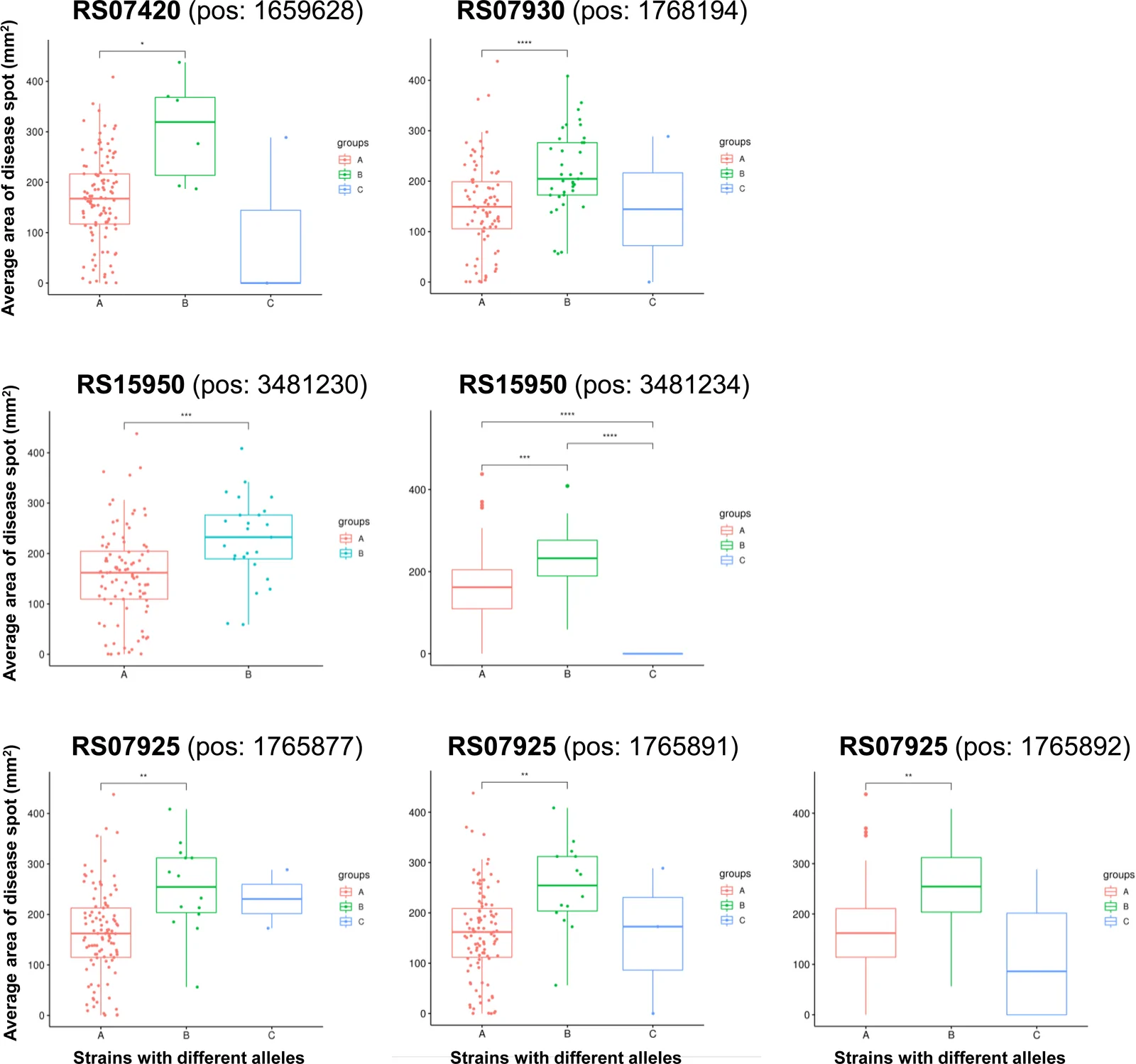
Comparison of virulence data for strains with different alleles in selected epistasis genes (loci). A, B, and C show the strains that have the same, different, or missing nucleotide bases compared to the reference genome at the indicated position. *, **, ***, and **** indicate *P* < 0.05, *P* < 0.01, *P* < 0.001, and *P* < 0.0001, respectively.

**FIG 8:**
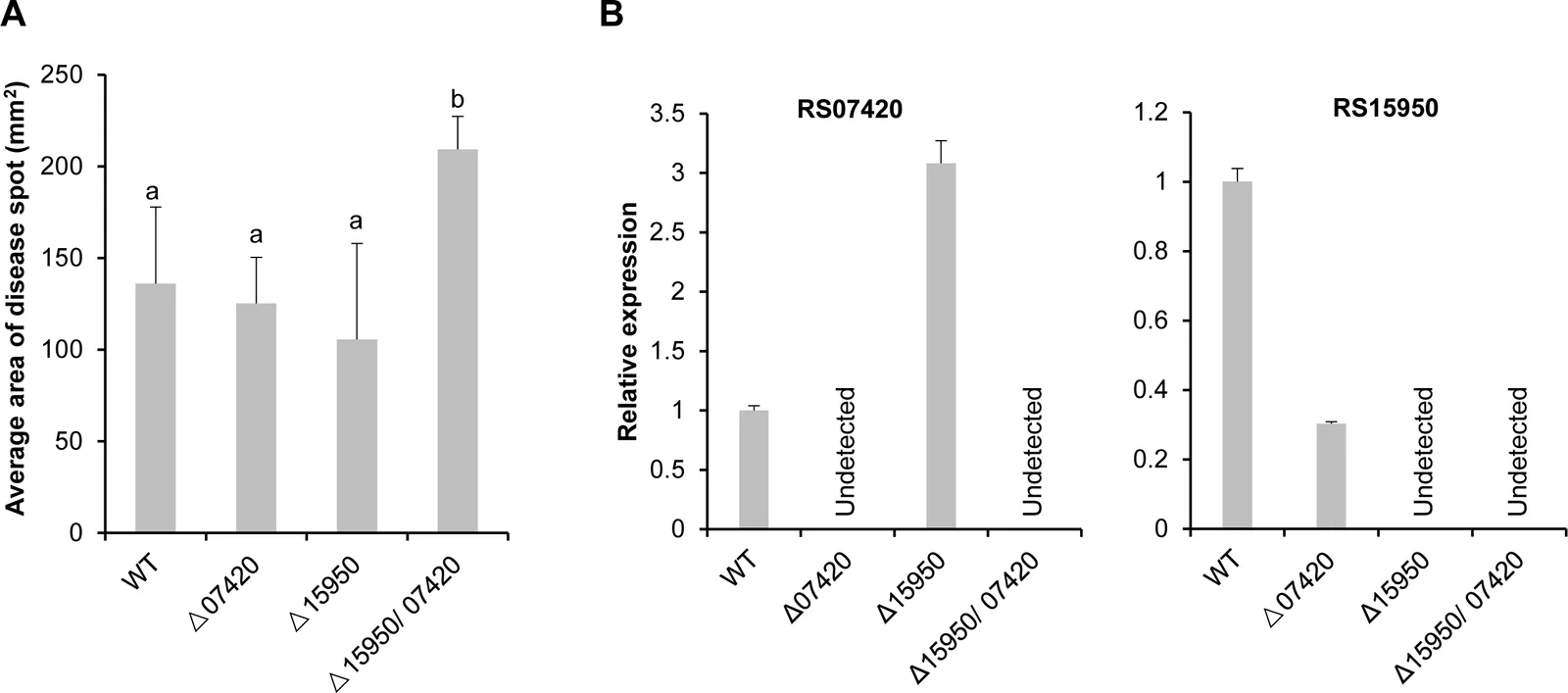
Validation of epistatic loci. (**A**) Virulence of single and double mutants of genes *RS07420* and *RS15950* (referred to as *Δ07420*, Δ*15950*, and Δ*15950*/*07420*), in addition to the WT strain. Virulence was evaluated from the average area of disease spots caused on Chinese cabbage *in vitro* (data are means ± SD, *n* = 6). Comparisons were performed among different strains using ANOVA analysis, and different letters indicate statistically significant differences (*P* < 0.05). (**B**) Relative gene expression of *RS07420* and *RS15950* in single and double mutants of genes *RS07420* and *RS15950* (referred to as Δ*07420*, Δ*15950*, and Δ*15950*/*07420*) compared to the WT strain. Each column shows the mean ± SD (*n* = 3).

To confirm the influence of the putative epistasis loci on phenotypes, virulence phenotypes were compared in the context of the loci from the three epistasis pairs that were targeted here. Variation in each locus significantly affected the virulence phenotype (Fig. 7), confirming that the epistasis loci play important roles in regulating *Pectobacterium* virulence. When the genes of pair BCS7_RS07420 (AraC family transcriptional regulator) and BCS7_RS15950 (multispecies: iron ABC transporter permease) were deleted separately or simultaneously, the virulence (as the average area of disease spot) of the single gene mutants Δ*07420* and Δ*15950* did not differ relative to the wild-type (WT) control (Po BCS7), while the double mutant Δ*15950*/*07420* exhibited greater virulence (Fig. 8A). Thus, the genes *RS07420* and *RS15950* might exert interactive effects on virulence, consistent with epistatic effects. In addition, the relative gene expression of *RS07420* and *RS15950* in mutants Δ*07420*, Δ*15950*, and Δ*15950*/*07420* was compared with the WT strain (Po BCS7) by the quantitative real-time PCR (qPCR) method. The results showed that *RS07420* exhibited higher expression in Δ*15950* than the WT control, while the expression of *RS15950* was lower in mutant Δ*07420* than that of the WT control (Fig. 8B), suggesting that the expression of *RS07420* and *RS15950* was affected by each other gene. The mutation of genes in each mutant was also verified to be undetected in the qPCR assay (Fig. 8B). Taken together, these results support the prediction that *RS07420* and *RS15950* have interacting epistasis effects.

## DISCUSSION

In this study, the phenotypic diversity of 120 *Pectobacterium* strains from China was compared based on their virulence and cellulase activity (i.e., pathogenicity-related phenotypes). In terms of virulence evaluation, an automated software program based on artificial intelligence was developed to measure lesion area, which facilitated accurate and rapid data collection for this phenotype. GWAS analysis for each of the phenotypes was performed using genome data for the 120 strains to better understand the genomic basis of the two phenotypes. GWAS analysis revealed a weak correlation between virulence and cellulase activity. Furthermore, epistasis loci were predicted based on the GWAS loci, with subsequent validation experiments confirming the genomic predictions. To the best of our knowledge, this is the first study to identify genetic associations among pathogenicity-related phenotypes in *Pectobacterium* using GWAS.

Accurate phenotypic data are key considerations in GWAS analysis. The discovery potential of GWAS could be weakened by phenotyping errors introduced by imprecise measurement methods (21). For example, small changes in the measurement of left ventricular ejection fractions (LVEFs) drastically impacted downstream genetic analyses, even within the same population (31). Introducing measurement noise as little as 7.9% can eliminate all significant genetic associations in GWAS with almost 40,000 individuals, and an increase of 1% in mean absolute error in LVEF had an equivalent impact on GWAS power as a decrease of 10% in the cohort sample size, suggesting that optimizing phenotyping precision is a cost-effective means to improve the power of genetic studies (31). Thus, the use of precise virulence data from *Pectobacterium* is important for GWAS analysis, although current lesion size measurement approaches are inadequate for these studies, including the lesion length detection method, the grid method, and the use of digital tools like ImageJ and Assess. The recent development of artificial intelligence image processing technology enabled the development of a new software program in this study that is based on deep learning and that can automatically measure lesion areas of soft rot. The software can segment lesion regions in <1 s and obtain true lesion area measurements for an image in <10 s. Furthermore, the novel method is more precise and convenient and faster than other methods. Thus, this software program drastically facilitates lesion measurements for investigating the soft rot disease of Chinese cabbage. Furthermore, the program could be modified to detect additional types of lesions caused by different diseases of various host plants, which would be especially useful in such studies.

Genes and their protein products rarely act independently, with transcriptional, translational, post-translational, and protein-protein interactions all playing important roles in dictating phenotypic characteristics (18). Interactions between genes, or epistasis, play an important role in controlling complex inheritance (30). In addition to being pathogens, phytopathogens can exist as benign epiphytes on plant surfaces or saprophytes in soil and water. Therefore, virulence factors and behaviors must be expressed in a coordinated manner for energy conservation, proper disease development, host defense evasion, and eventual spread. Therefore, a regulated global virulence regulatory network is essential for a phytopathogen’s survival, such as *Pectobacterium* (32). Consistently, our analysis predicted the existence of epistasis in virulence-associated phenotypes through population genome analysis, providing gene resources that might have interacting roles in the virulence of *Pectobacterium* from a global perspective.

The members of the AraC family of transcriptional regulators are widespread among Gram‐negative and Gram‐positive bacteria (33). They generally activate genes involved in virulence, stress responses, or carbon metabolism (34), with the emphasis that they commonly play crucial virulence regulation roles in association with T3SSs in animal and plant bacterial pathogens (32). However, *P. carotovorum* (previously *P. carotovorum* subsp. *carotovorum*) does not use AraC-like proteins to regulate its T3SS (32), and, to our knowledge, the function of the AraC transcriptional regulator in *Pectobacterium* has not been reported yet. In our prediction, the AraC family transcriptional regulator (BCS7_RS07420) exerted epistatic effects with three other virulence-related genes, including TonB-dependent receptor (BCS7_RS07925), iron-siderophore ABC transporter substrate-binding protein (BCS7_RS07930), and iron ABC transporter permease (BCS7_RS15950) (Table 1). Meanwhile, epistasis effects between the AraC family transcriptional regulator (BCS7_RS07420) and iron ABC transporter permease (BCS7_RS15950) were also supported by experimental evidence (Fig. 7 and 8). The TonB-dependent receptor is related to the transport of outer membrane nutrients in Gram-negative bacteria, including siderophores, vitamin B12, and saccharides (35), thereby contributing to pathogen virulence. ABC transport systems, including substrate-binding proteins, are involved in the uptake of various critical nutrient substrates into cells, including metal ions, sugars, and vitamins that can help maintain the virulence of bacteria and pathogens (36). The three genes predicted to have epistasis effects with the AraC transcriptional regulator are all possibly related to nutrient acquisition, via iron transport, for example. Taken together, our results provide new clues as to how the AraC transcriptional factor might function in the virulence of *Pectobacterium*, e.g., by regulating nutrient acquisition via TonB-dependent receptors and iron ABC transporter-related proteins. Their interactions and roles in the virulence of *Pectobacterium* should be further investigated.

The virulence of *P. carotovorum*, *P. brasiliense*, and *P. odoriferum* was determined *in vitro* based on lesion areas of Chinese cabbage, revealing a lack of significant differences among the three species. The result is consistent with a previous virulence assessment (37) based on the diameter of rotting tissue in potato tubers caused by the three pathogens. However, the results contrast with those of our previous study, wherein *P. odoriferum* (previously identified as *P. carotovorum* subsp. *odoriferum*) exhibited significantly higher virulence than *P. brasiliense* and *P. carotovorum* strains (38). It remains unclear why these results are inconsistent, but it could potentially be due to variability among strains used in different studies and/or differences in virulence assessment approaches. The maceration ability of *Pectobacterium* strains is notably associated with their host of origin (38). Thus, host origin requires further investigation to understand if it contributes to the varying results reported here and elsewhere.

In summary, GWAS were used in this study to identify the determinants of *Pectobacterium* pathogenicity and provide new insights into their mechanisms of pathogenicity. The results provide new targets for further identifying *Pectobacterium* virulence factors and promoting their control.

## MATERIALS AND METHODS

### Bacterial strains, plasmids, and culture conditions

A total of 120 *Pectobacterium* strains were used for GWAS analysis (Table S1), including 26 *P. carotovorum* (formerly *P. carotovorum* subsp. *carotovorum*) strains, 47 *P. brasiliense* (formerly *P. carotovorum* subsp. *brasiliense*) strains, and 46 *P. odoriferum* (formerly *P. carotovorum* subsp. *odoriferum*) strains, in addition to 1 *P. versatile* (formerly *Candidatus* P. maceratum) strain and Ecc71 (initially identified as *P. carotovorum* subsp. *carotovorum* and later as *Candidatus* P. maceratum). The strains were previously identified as subspecies of *P. carotovorum* and are closely related. Other bacterial strains and plasmids used in this study are shown in Table S8. The rifampicin-resistant strain *P. odoriferum* BCS7 was used as the WT strain for functional validation of mutants. The strains were grown on Luria-Bertani (LB) broth or LB agar plates. The *Pectobacterium* strains were grown overnight at 28°C, while *Escherichia coli* strains were grown overnight at 37°C. Antibiotics were used, when required, at the following concentrations (in μg/mL): rifampin (Rif), 100; kanamycin (Km), 50; gentamicin, 50; and streptomycin, 50.

### Genome sequences, GWAS, and epistasis analysis

Publicly available genome sequences for 30 strains were retrieved from the GenBank database (8). The genome sequences for the other 90 strains were generated in this study and deposited into the NCBI SRA database under BioProject accession number PRJNA992924. Total genomic DNA was extracted from overnight cultures grown in LB medium using an EasyPure Bacteria Genomic DNA Kit (Transgen Biotech, China) following the manufacturer’s guidelines. The genomes were sequenced on the Illumina NovaSeq 6000 platform to generate 150-bp paired-end reads. Clean reads were aligned to the reference genome of Po BCS7 (CP009678.1) and subjected to SNP identification using the samtools aligner (v.1.1, http://github.com/samtools/) and the GATK (v.4.2.0.0, https://gatk.broadinstitute.org/hc/en-us) software program. SNPs were identified as two alleles with max missing >95% and MAF >0.05 using the Vcftools (v.0.1.13) program (39). A total of 230,289 SNPs and 4,506 indels were identified. SNP annotation was conducted using the snpEFF v.5.0e program (40) and the assembly version GCF_000769535.1_ASM76953v1_genomic.gff. A phylogenetic tree was constructed using the final SNP data set with the RAxML program (v.8.2.12) and 100 bootstrap replicates.

#### Processing of phenotype data

Lesion area data exhibited a normal distribution and were thus directly used for GWAS analysis after eliminating individual outliers. However, the cellulase activity data did not exhibit a normal distribution, necessitating a COX-BOX conversion before subsequent GWAS analysis.

#### GWAS

Three models, including a linear model (LM), a linear model considering population structure, and a linear model considering population structure and genetic relationships (QK), were used for the GWAS analysis of each phenotype. The best model was chosen for the GWAS analysis for each phenotype based on inflation factors that were closest to 1. Specifically, the QK model was used for the virulence phenotype analysis, while the LM model was used for the cellulase activity phenotype analysis. The genome-wide statistical significance threshold for GWAS was *P* < 0.001. Statistically significant associations were visualized using a Manhattan plot (40). For epistatic analysis, a new variable was initially generated by merging two markers. The association between the new variable and phenotype was then tested using the FASTmrEMMA software package (41) that accounts for population structure and kinship relationships. To ensure statistical accuracy, multiple testing corrections were applied using a false discovery rate analysis (41).

### Software development for automated measurements of soft rot lesions on Chinese cabbage

The automated lesion measuring software program (termed “Lesionsurvey”) was developed using the Visual Studio 2015 platform based on Microsoft Foundation Classes and was executable on Windows x64 systems. The Convolutional Architecture for Fast Feature Embedding deep learning framework was used for the program. The software function modules included image input and display, resolution calibration, automatic extraction of lesion areas, manual adjustment of areas, area calculations, displaying results, and exporting results. The core module of the software computed the segmentation of lesions using the fully convolutional network semantic segmentation model for deep learning. The model was trained on many manually labeled images to obtain parameters with good segmentation effects. The module then ran the segmentation model to output lesion areas after loading the image.

### Pathogenicity phenotype assessment

The pathogenicity of *Pectobacterium* strains and the resultant maceration of Chinese cabbage petioles were determined *in vitro*. The inoculation method was nearly identical to that described by Li et al. (38), with minor modifications. Briefly, 3 µL (for GWAS analysis) or 5 µL (for mutant validation) of bacterial suspensions (2 × 10^8^ CFU/mL) of each strain was inoculated per site into Chinese cabbage with 2.5 mm deep inoculations. The rot area for each petiole was measured 24 h later using the Lesionsurvey software described above. Six or nine replicate petioles were established for the pathogenicity assessment of each strain for the GWAS or mutant analysis (three petioles were used per petri dish). All experiments were replicated at least twice, and similar trends were observed among replicates. Cellulase activity was assessed using the Ostazin brilliant red-cellulose method, as previously described (42). In brief, supernatants of overnight cultures of each strain with an OD600 = 2.5 were obtained by centrifugation. Then, 50 µL of each supernatant sample was incubated with 200 µL of 2.5 mg/mL carboxymethyl cellulose in 0.025 M sodium phosphate buffer (pH = 7) and subjected to the following measurement steps. Three replicates were included for each strain.

### KEGG enrichment analysis

Genes identified within the GWAS using Po BCS7 as the reference genome were annotated against the KEGG database, and KEGG enrichment analysis was subsequently performed using the tool on Omicshare (https://www.omicshare.com/tools/) under default parameters.

### Construction of *P. odoriferum* mutants

Single gene depletion mutants of *P. odoriferum* BCS7, including Δ*07420* and Δ*15950*, were constructed by homologous recombination, as previously described (43), but with some modifications. Briefly, the upstream and downstream regions of target genes were amplified by PCR using relevant primers (Table S9). A Kan cassette, amplified from pET30a with a Lox site added, was ligated with the two fragments and cloned into the pEX18Gm vector using the In-Fusion HD Cloning Kit (Takara, China) to generate a recombinant vector. The vector was then transferred into Po BCS7 by conjugation using *E. coli* S17-1. To select strains with gene deletions, transconjugants were plated on LB containing 10% sucrose, Rif, and Km, followed by confirmation with PCR. Depletion mutant Δ*15950*/*07420* of double genes *RS07420* and *RS15950* (Che et al., unpublished data) was obtained by removing Kan via Cre recombinase activity in a single gene mutant of Δ*15950* through conjugation with *E. coli* S17-1 harboring pEX18Gm-Cre first. The Kan removal mutant strain was then used as the recipient to construct a transconjugant with depletion of the second gene, *RS07420*. The plasmids and strains used in this study are shown in Table S8, while the PCR primers used for strain construction are shown in Table S9.

### Quantitative real-time PCR assays of gene expression

Relative gene expression of *RS07420* and *RS15950* in single and double mutants of genes *RS07420* and *RS15950* (referred to as Δ*07420*, Δ*15950*, and Δ*15950*/*07420*) compared to the wild-type strain *P. odoriferum* BCS7 was tested. The *Pectobacterium* strains were cultured overnight, and a pellet was collected by centrifugation at 8,000 rpm and 4°C for 1 min. Total RNA was extracted from the cells using the Spin Column Bacteria Total RNA Purification Kit (Sangon Biotech, China) and treated with DNase I. cDNA was then synthesized from the total RNA and subjected to qPCR using the TB Green Premix Ex Taq II (Tli RNaseH Plus) (TaKaRa, China), as previously described (44). RecA was used as the qPCR reference gene. The fold change in the relative gene expression of *RS07420* and *RS15950* in samples of Δ*07420*, Δ*15950*, and Δ*15950*/*07420* compared with the WT strain was analyzed using the 2^−∆∆CT^ method (45). The qPCR primers used in the assays are shown in Table S9. Experiments were repeated twice, with three technical replicates for each experiment.

## Data Availability

All data supporting the research findings of this study are included within the article and in the supplemental material. Raw data of the newly generated genome sequences in this study were deposited into the NCBI SRA database under BioProject ID PRJNA992924 with the accession numbers SRR25209396 to SRR25209485 (https://www.ncbi.nlm.nih.gov/Traces/study/?acc=PRJNA992924&o=acc_s%3Aa). The Lesionsurvey source code, test data set, and documentation for running software can be found at https://github.com/afternoonzhou/Lesionsurvey.git.

## SUPPLEMENTAL MATERIAL

Legends of Tables S1 to S9 spectrum.01764-23-s0001.pdf Supplemental legends.

Tables S1 to S9 spectrum.01764-23-s0002.xlsx Supplemental tables.

ASM does not own the copyrights to Supplemental Material that may be linked to, or accessed through, an article. The authors have granted ASM a non-exclusive, world-wide license to publish the Supplemental Material files. Please contact the corresponding author directly for reuse.
