# Supplementary material for: A genome-wide association study reveals that epistasis underlies the pathogenicity of Pectobacterium
Source: Microbiol Spectr. 2023 Sep 15;11(5):e01764-23. doi: 10.1128/spectrum.01764-23 (PMC10580964; doi:10.1128/spectrum.01764-23)
Supplement: Legends of Tables S1 to S9 — Supplemental legends. [file spectrum.01764-23-s0001.pdf]

Supplemental Material

Table S1. Information for the 120 *Pectobacterium* strains used in this study.

Table S2. Genome-wide association signals for the virulence phenotype.

Table S3. Genome-wide association signals for the cellulase activity phenotype.

Table S4. Epistatic loci identified for the virulence phenotype.

Table S5. Epistatic loci identified for the cellulase activity phenotype.

Table S6. Epistatic loci annotated as missense mutation for the virulence phenotype.

Table S7. Epistatic loci annotated as missense mutation for the cellulase activity phenotype.

Table S8. Other bacterial strains and plasmids used in this study.

Table S9. Primers used in this study.
